# Using lipidomics to reveal details of lipid accumulation in developing seeds from oilseed rape (*Brassica napus* L.)^☆^

**DOI:** 10.1016/j.bbalip.2017.12.010

**Published:** 2018-03

**Authors:** Helen K. Woodfield, Amaury Cazenave-Gassiot, Richard P. Haslam, Irina A. Guschina, Markus R. Wenk, John L. Harwood

**Affiliations:** a School of Biosciences, Cardiff University, Cardiff CF10 3AX, UK; b Department of Biochemistry, National University of Singapore, Singapore 117587, Singapore; c Singapore Lipidomics Incubator (SLING), Life Sciences Institute, National University of Singapore, Singapore 117456, Singapore; d Department of Plant Sciences, Rothamsted Research, Harpenden, Hertfordshire AL5 2JQ, UK

**Keywords:** Developing oilseed rape, *Brassica napus* (L), Lipidomics, Lipid accumulation, Regulation of synthesis

## Abstract

With dwindling available agricultural land, concurrent with increased demand for oil, there is much current interest in raising oil crop productivity. We have been addressing this issue by studying the regulation of oil accumulation in oilseed rape (*Brassica napus* L). As part of this research we have carried out a detailed lipidomic analysis of developing seeds.

The molecular species distribution in individual lipid classes revealed quite distinct patterns and showed where metabolic connections were important. As the seeds developed, the molecular species distributions changed, especially in the period of early (20 days after flowering, DAF) to mid phase (27DAF) of oil accumulation. The patterns of molecular species of diacylglycerol, phosphatidylcholine and acyl-CoAs were used to predict the possible relative contributions of diacylglycerol acyltransferase (DGAT) and phospholipid:diacylglycerol acyltransferase to triacylglycerol production. Our calculations suggest that DGAT may hold a more important role in influencing the molecular composition of TAG. Enzyme selectivity had an important influence on the final molecular species patterns.

Our data contribute significantly to our understanding of lipid accumulation in the world's third most important oil crop.

## Highlights

• Lipidomic analysis of developing rapeseed seeds is reported
• Results show distinct differences between lipid classes
• Changes in molecular species distributions were found during development
• The data were used to evaluate the contribution of different synthetic pathways

## 1 Introduction

Plant oils are major agricultural commodities with a current market value of over US$120 billion [1]. Moreover, demand for such oils has been increasing at about 5% per year for the last five decades [2]. So far, improvements in productivity and sowing larger areas have managed to keep pace with demand. However, finite agricultural land, increasing populations and more widespread use of crops for renewable chemicals/biofuels suggest that plant oils will soon be in short supply [3].

Although the basic characteristics of oil synthesis in terms of enzymology are well understood in the model species Arabidopsis [4], [5], [6], less is known about crop plants and, in particular, our knowledge of its regulation is much less secure [7]. More detailed research has revealed subtleties of the biosynthetic process, with new, relevant enzymes discovered [see [3]], multiplicity of pathways demonstrated [8] and compartmentation of triacylglycerol accumulation made evident [9].

We have studied the regulation of oil accumulation in crops, partly by the application of flux control analysis [10], [11], [12], [13]. These experiments have revealed important overall characteristics of the process but often could not delineate some of the details such as the subsidiary flux of fatty acids from the basic Kennedy pathway into and out of phosphatidylcholine (PC), either in terms of polyunsaturated fatty acid production [14], [15] or via phospholipid:diacylglycerol acyltransferase (PDAT) [16]. To elucidate some of these details and to further our knowledge of regulation, we have utilised lipidomics. Since modern lipidomics is useful for identifying metabolic networks and testing hypotheses about control [17] we have used it here to elucidate details of metabolism and further our knowledge of regulation.

The overall process to synthesise accumulating triacylglycerol (TAG), ultimately in seed lipid droplets [18], begins with the de novo synthesis of fatty acids in plastids [4], [19], [20]. After seven basic cycles of 2 carbon additions, palmitoyl-ACP is produced which can be hydrolysed to release palmitic acid or elongated using β-ketoacyl-ACP synthase II (KASII) to give stearoyl-ACP. A very active Δ9-desaturase in plastids [21] ensures that most plants produce a mixture of palmitic and oleic acids (in about a 1:4 ratio) as end products of de novo synthesis. Transport of fatty acids from the plastid and their addition to the cytosolic acyl-CoA pool has been discussed recently [22]. In addition, the role of acyl-CoA binding proteins (ACBPs) in this process and subsequent participation in fatty acid modification is largely unresolved [23]. Lipid assembly via the Kennedy pathway [24] and ancillary reactions, in the endoplasmic reticulum, has been well discussed [3], [4], [5]. Overlaid on this detailed biochemistry is work which has revealed overall control of carbon flux such as by WRI1 (WRINKLED1) [25] or FUSCA3 transcription factors [26]. In addition, experiments have used a push/pull engineering strategy [27] where carbon is channelled into lipid biosynthesis and the end stages of oil accumulation, such as diacylglycerol acyltransferase (DGAT) increased to prevent build-up of intermediates. This has been applied to soybean seeds [28] and also to other tissues [e.g. [29]].

Oilseed rape is one of the major world oil crops, yielding about 12% of total world oil market [30]. It is the major Northern European and Canadian oil crop and, because of its close relation to Arabidopsis and ease of genetic manipulation, has been extensively modified to produce renewable chemicals or speciality fatty acids [31], [32]. *Brassica napus* has two distinct groups of cultivars – the low erucate (LEAR) and high erucate (HEAR) types (where erucic acid is *cis*-13-docosenoic acid (22:1)). HEAR has mainly industrial uses, while LEAR is more extensively grown (called Canola in Canada) and is used predominantly for human consumption and animal feed.

Our previous biochemical experiments identified diacylglycerol:acyl-CoA transacylase (DGAT) as an important regulatory enzyme in *B*. *napus* for carbon flux into oil [33], [34] and over-expression of DGAT was shown to increase TAG accumulation [35] in both greenhouse experiments and field trials [36]. Further experiments have given detailed information about flux control of oil synthesis in this crop [12]. In particular (and in contrast to other oil crops that we studied [7]), our experiments in oilseed rape indicated that lipid assembly exerted more control over oil accumulation than fatty acid biosynthesis [12], [35].

While the classic Kennedy pathway underpins lipid assembly during TAG biosynthesis [24], in plants extra enzyme steps are important [3], [9], [14]. In particular, PDAT [16] provides an acyl-CoA-independent source of fatty acids for TAG assembly. However, the relative importance of PDAT in different oil crops is currently uncertain [see 9]. Nevertheless, PDAT can be said to complement activity of DGAT [37] at least in some plants e.g. Arabidopsis. Measurements of DGAT and PDAT in oilseed rape showed that PDAT has rather little activity [12]. However, these measurements were made in vitro under optimal enzymatic conditions and may not reflect accurately the situation in vivo. Likewise, genetic manipulation of DGAT and/or PDAT creates artificial stresses. Moreover, although transcriptional profiling of *B*. *napus* showed that expressed sequence tag (EST) abundance for DGAT is much higher than for PDAT [38], this may not translate directly into enzyme activity. It was partly to address these questions that we have used lipidomics in the present study. With improvements in mass spectrometry over the last two decades, the use of lipidomics has gathered pace [39], [40], [41], [42]. Such techniques have been applied to plant tissues [43], [44], [45].

As part of our studies to elucidate details of the regulation of lipid accumulation in oilseed rape, we have used a range of techniques including basic biochemistry [33], [34], use of transgenic lines [35], [36], flux control analysis [12], [35] and MALDI analysis [46]. To extend and complement these studies we have now applied detailed mass spectrometric (MS) analysis to developing oilseed rape seeds. The lipidomic data has provided important information about lipid metabolism during oil accumulation that reveals details of the biochemical pathways and enzymology involved.

## 2 Materials and methods

### 2.1 Materials

*Brassica napus* cv. Westar seeds were a kind gift from Professor R.J. Weselake (University of Alberta, Edmonton, Canada). Seeds were germinated in multipurpose compost (MS Levington compost) mixed (3:1) with fine sand. Seedlings were placed in seed trays and grown for 10 days before transplanting individually into 8-inch. pots. Growth was in a greenhouse with a temperature of 23 °C and supplemental lighting to maintain a light intensity of 250 μmol m^− 2^ s^− 1^ with a 16 h light period. Flowers were hand pollinated and tagged on the day of flower emergence.

### 2.2 Lipid extraction and standard analysis

Siliques were harvested at 20, 27 and 35 days after flowering (DAF), representing early, mid and late phases of lipid accumulation in oilseed rape [47]. Ten seeds (~ 45 mg fresh weight) from different siliques were harvested for each biological repeat. Tissues were treated with isopropanol at 70 °C for 30 min followed by a two-phase extraction procedure [48] shown to be efficient (> 98%) for the lipids analysed from plant tissues. The washed lower phase was taken to dryness under nitrogen, dissolved in chloroform and stored at − 20 °C under nitrogen until further analysis.

Non-polar lipids were separated by thin layer chromatography (TLC) using a solvent mixture of hexane/diethylether/acetic acid (80:20:1, by vol.). Polar lipid classes were separated by 2-dimensional TLC using chloroform/methanol/water (65:25:4, by vol.) in the first direction and chloroform/acetone/methanol/acetic acid/water (50:20:10:10:5, by vol.) in the second. Spraying with 0.2% (w/v) 8-anilino-1-naphtholenesulphonic acid in anhydrous methanol and viewing under U.V. light [49] was used to reveal lipid bands. Standards were obtained from Nu-Chek Prep. Inc., Elysian, MN, USA.

For analysis of acyl composition, individual lipid bands were separated from TLC plates and fatty acid methyl esters (FAMEs) were prepared by acid-catalysed methylation (2.5% H_2_SO_4_ in methanol). An internal standard of nervonic acid (*cis*-15-tetracosenoic acid, 24:1) was used. FAMEs were separated on a 30 m × 0.25 mm i.d. capillary column (Elite 225, Perkin-Elmer, Normalk, CT, USA) using a Clarus 500 gas chromatograph with a FID detector [50]. FAMEs were routinely identified by comparison of retention times with those of a GC-411 standard (Nu-Chek) with identities confirmed by GC–MS (see [51]). Perkin-Elmer TotalChrom software was used for data acquisition and calculations. These methods were used to calculate amounts of the different lipid classes from oilseed rape (on a fatty acid basis).

### 2.3 Lipidomic analysis

Dried lipid extracts were re-suspended in 1 ml chloroform:methanol (2:1; v/v) and further diluted five times with the same solvent, then mixed 1/1 (v/v) with the internal standard solution (IS) before separation by LC/MSMS. A quality control (QC) sample was prepared by pooling 20 μl of each sample together. The IS solution was prepared by dilution of stock solutions of dimyristoyl phosphatidylcholine (PC 28:0, final concentration in IS solution: 0.738 pmol/ml), dimyristoyl phosphatidylethanolamine (PE 28:0, final concentration in IS solution: 0.785 pmol/ml), dimyristoyl phosphatidic acid (PA 28:0, final concentration in IS solution: 0.813 pmol/ml), didodecanoyl glycerol (DAG 24:0, final concentration in IS solution: 4.379 pmol/ml), and deuterated-trihexadecanoyl glycerol (d5-TAG 48:0, final concentration in IS solution: 24.618 pmol/ml). All standards were purchased from Avanti Polar Lipids (Alabaster, USA).

LC separation was undertaken on an UHPLC 1260 (Agilent, Santa Clara, USA) using hydrophilic interaction liquid chromatography (HILIC) for phospholipids (PC, PE and PA) and reverse phase (RP) for non-polar lipids (DAG and TAG). HILIC conditions were: Injection volume 2 μl, mobile phase A: 95% acetonitrile/5% 25 mM ammonium formate pH 4.6, mobile phase B: 50% acetonitrile/50% 25 mM ammonium formate pH 4.6, column: Kinetex HILIC, 2.1 × 150 mm, 2.6 μm, column temperature: 30 °C, flow rate 0.5 ml/min, gradient: 0 min: 0.1% B, 6 min: 75% B, 7 min:90% B, 7.1 min 0.1%B, 10.1 min: end of run. RP conditions were_:_ Injection volume 2 μl, mobile phase A: 40% acetonitrile/60% 10 mM aqueous ammonium formate, mobile phase B: 90% isopropanol/10% 10 mM ammonium formate in acetonitrile, column: Zorbax Eclipse Plus C18, 2.1 × 50 mm, 1.8 μm, column temperature: 40 °C, flow rate 0.4 ml/min, gradient: 0 min: 20% B, 2 min: 75% B, 6 min: 100% B, 9 min:100% B, 9.01 min 20%B, 11 min: end of run. All solvents were LC-MS grade from Fischer (Pittsburg, USA).

MS/MS analysis was undertaken on a 6460 triple quadrupole (Agilent, Santa Clara, USA) with an electrospray ionisation source. Parameters were as follows: gas temperature 300 °C, gas flow 5 L/min, nebulizer 45 psi, capillary 3500 V. DAG and TAG were analysed with positive ionisation as ammonium adducts, using both single ion monitoring (SIM) for sum composition (e.g. TAG 54:3) and multiple reaction monitoring (MRM) transitions to neutral loss of a single fatty acyl (e.g. TAG 16:0/18:2/18:2 to 18:2 neutral loss). Although only single fatty acyl MRM transitions were monitored, TAG species are reported with three fatty acyls (e.g. TAG 16:0 18:2 18:2) based on preliminary LC-high-resolution-MS/MS data acquired on a quadrupole-time of flight instrument. Details of the MS/MS data are given in Supplementary Table 1. PC, PA and PE were analysed using MRM transitions to headgroup and fatty acid fragments using both positive and negative ionisation. A full list of SIM and MRM, including parameters and adducts, can be found in Supplementary Table 2.

After instrument stabilisation with six injections of a QC sample, instrument stability was monitored by injecting a QC sample and a blank every six sample injections. QC injections were used to calculate coefficient of variations (CoV) for each SIM and MRM. All reported species had CoV lower than 25%.

### 2.4 Lipidomic data processing

Relative quantification data were extracted using Agilent MassHunter Quantitative Analysis (QQQ) software. The data were manually curated to ensure that the software integrated the right peaks. Areas under curve (AUC) of the extracted ion chromatograms peaks for each SIM/MRM transition were extracted to Excel. A correction factor was applied to account for the multiplicity of fatty acids in some molecular species of TAG (e.g. TAG 18:1 18:1 18:1). For HILIC, isotopic correction was performed on AUC of the phospholipids according to [52]. AUCs were normalised either to that of internal standards and to sample weight, or to total intensity in a lipid class. Statistical analysis was performed using a generalised linear model with gaussian distribution.

### 2.5 Acyl-CoA profiling

Seeds from siliques harvested at 20, 27 and 35 days after flowering (DAF) were frozen in liquid nitrogen and then extracted after [53] for reverse-phase LC with electrospray ionisation tandem mass spectrometry (multi reaction monitoring; using a SCIEX 4000QTRAP instrument) in positive ion mode. LC-MS/MS MRM analysis followed the methods described in [54]. Acyl-CoA were separated using an Agilent 1200 LC system; Gemini C18 column, 2 mm inner diameter, 150 mm with 5 μm particles. For the purpose of identification and quantification, standard acyl-CoA esters with acyl chain lengths from C14 to C20 were synthesised from free acids or lithium salts (Sigma-Aldrich, St Louis, Missouri, USA). Heptadecanoyl coenzyme A (ammonium salt) was used as an internal standard in each analytical run. Retention times also confirmed using acyl-CoA standards from Avanti.

## 3 Results

### 3.1 Analysis of lipid molecular species at a midpoint of lipid accumulation

In order to evaluate the lipidomic methodology we analysed molecular species of major lipid classes at a mid-point of lipid accumulation in oilseed rape. Previous studies [47] and our own with *B*. *napus* cv. Westar [46] have shown that the rapid period of lipid accumulation was in the period 20–35 DAF. A mid-point at 27 DAF was selected and analyses made of the accumulating TAG, the important metabolic intermediates, DAG, PA, and PC, as well as the second most prevalent seed phosphoglyceride (PE) as a comparator.

**Fig. 1:**
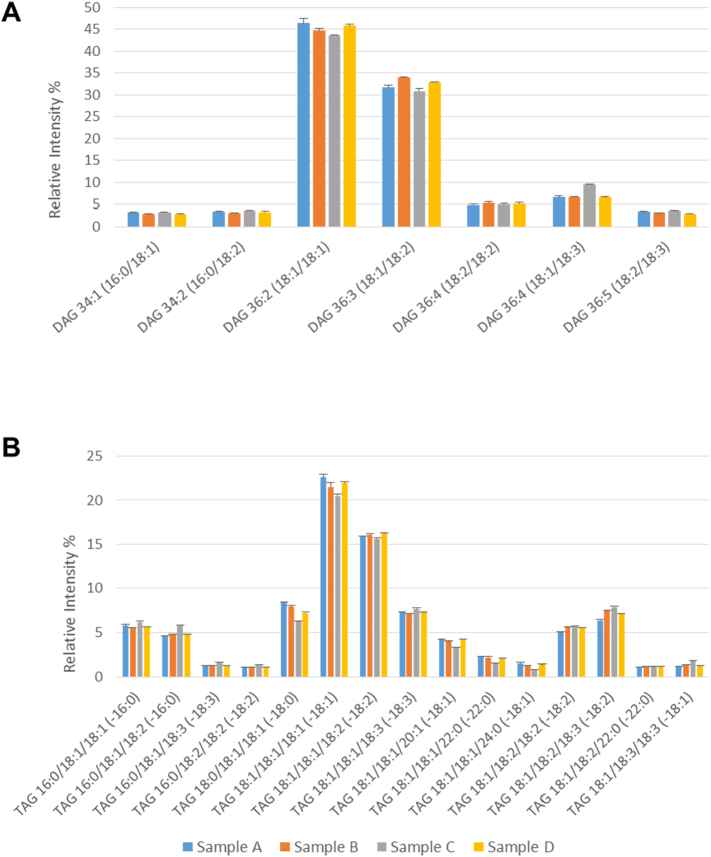
Analysis of acyl glycerols at a mid-point (27 DAF) of lipid accumulation in oilseed rape. Diacylglycerol (A) and triacylglycerol (B) molecular species were analysed by multiple reaction monitoring (MRM) using a triple quadrupole instrument after separation of the lipid classes by reverse phase HPLC as described in Materials and methods. Four individual biological samples are depicted, each of which was analysed with technical triplicates (means ± SD shown). Major molecular species (> 1% total) are detailed here, with further information on minor species listed in Supplementary Tables 3 and 4. The fatty acid abbreviation in parenthesis refers to the MRM transition (neutral loss of a specified fatty acid) used for the measurement. At the mid-point of lipid accumulation (27 DAF) the amounts of diacylglycerol and triacylglycerol were 0.66 ± 0.24 and 72.5 ± 0.6 μg fatty acid/mg FW embryo, respectively, as measured by TLC/GLC.

After separating the acyl glycerols by reverse phase HPLC, the main molecular species of DAG and TAG are shown in Fig. 1A and B, respectively. Their full analyses are given in Supplementary Tables 3 and 4. Fig. 1 shows four separate biological repeats and it can be seen that these were reproducible for both DAG and TAG.

For DAG, the main molecular species corresponded to 36:2 (18:1/18:1) and 36:3 (18:1/18:2) (Fig. 1A). The molecular species 36:2 and 36:3 represented around 45% and 33% of the total, respectively, as might be expected given the overall lipid composition of oilseed rape oil [2]. Less prevalent, though still significant, species were those containing greater amounts of polyunsaturated fatty acids (36:4, 36:5) or palmitic acid (34:1, 34:2).

Four separate biological samples were analysed for their TAG molecular species composition (Fig. 1B, Supplementary Table 4). Again, there was good reproducibility with only one of four samples showing moderate deviation from the others. This was in spite of inherent biological variability and the significant number of steps from harvesting to analysis. Two TAG species were prominent with 54:3 (18:1/18:1/18:1) being > 54:4 (18:1/18:1/18:2). As seen with the DAG molecular species, other prominent molecular species were usually those containing additional unsaturation (54:5, 54:6) or with palmitate (52:2, 52:3). However, four additional TAG molecular species contained stearate (54:2 (18:0/18:1/18:1), 7%) or very long chain fatty acids (VLCFAs) such as gondoic (*cis*-11-eicosenoic acid, 20:1) (56:3 (18:1/18:1/20:1), 4%), behenic (docosanoic acid, 22:0) (58:2 (18:1/18:1/22:0), 3%) and lignoceric (tetracosanoic acid, 24:0) (60:2 (18:1/18:1/24:0), 2%) acids. It was notable that erucate was not detected in the TAG species analysed for cv. Westar at 27 DAF.

**Fig. 2:**
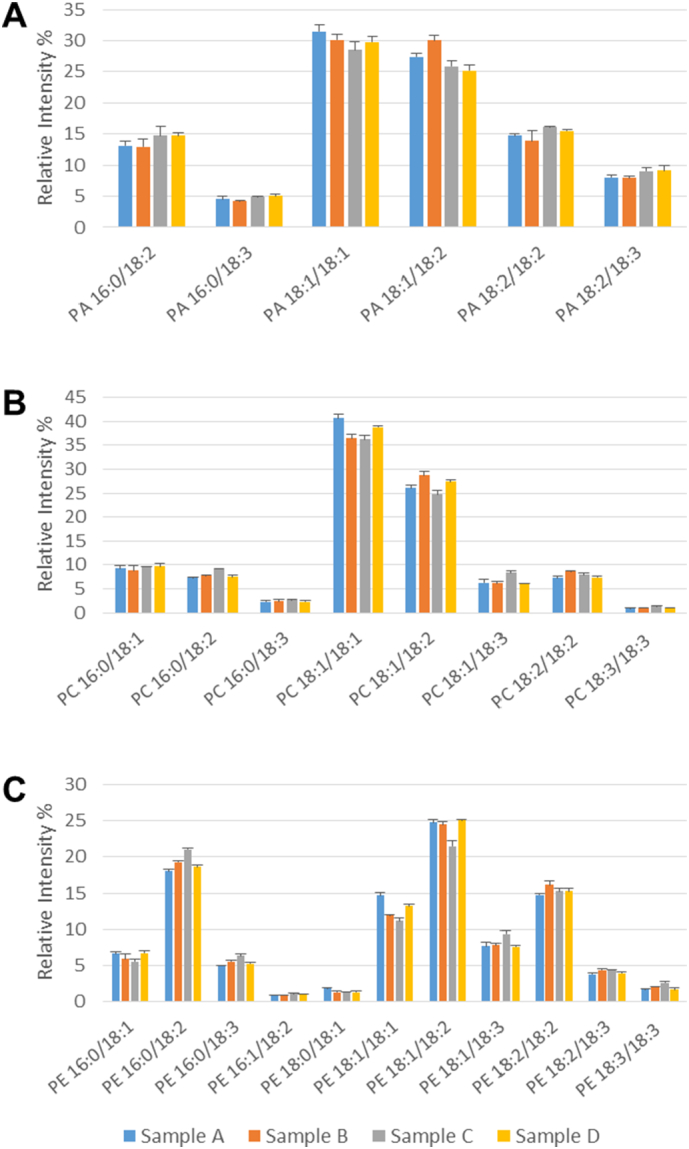
Analysis of phosphoglycerides at a mid-point (27 DAF) of lipid accumulation in oilseed rape. Phosphatidate (A), phosphatidylcholine (B) and phosphatidylethanolamine (C) molecular species were separated by hydrophilic interaction liquid chromatography (HILIC) and analysed by multiple reaction monitoring as detailed in Materials and methods. Four individual biological samples are shown, each of which was analysed with technical triplicates (means ± SD shown). Individual molecular species of 1% or greater abundance are detailed with full information of all species detected given in Supplementary Tables 5–7. The relative amounts of the PA, PC and PE at 27 DAF were 0.32 ± 0.14, 2.37 ± 0.17 and 0.38 ± 0.03 μg fatty acid/mg FW embryo, respectively, as measured by TLC/GLC.

The key metabolic intermediate, phosphatidate (PA), was analysed and the main species are shown in Fig. 2A with full data in Supplementary Table 5. As for the DAG results (Fig. 1), the main molecular species were 18:1/18:1 and 18:1/18:2 at about 30% and 27%, respectively. However, 16:0/18:2 and 18:2/18:2 were also significant, at 14–15% which was higher than for DAG (Fig. 1A). Other notable species were 18:2/18:3 (9%), 16:0/18:3 (5%) (Fig. 2A) and 16:1/18:1 (1%).

The major molecular species of PC are shown in Fig. 2B with full data in Supplementary Table 6. The major species were 18:1/18:1 (38%) and 18:1/18:2 (27%). As for DAG and PA, other significant species were those containing palmitate (16:0/18:1 (9%), 16:0/18:2 (8%), 16:0/18:3 (3%)) or increased unsaturation (18:1/18:3 (7%), 18:3/18:3 (9%)) (Fig. 2B, Supplementary Table 6).

Because PC is intimately involved in TAG synthesis in oil crops [1], [14], we also analysed phosphatidylethanolamine (PE) which is synthesised by a similar pathway to PC [24], [55] but neither participates directly in fatty acid desaturation [56] nor is involved in TAG accumulation [5], [57]. The pattern of molecular species (Fig. 2C) was noticeably distinct from PC (Fig. 2B). Full data is shown in Supplementary Table 7. In order of abundance the molecular species were 18:1/18:2 (24%), 16:0/18:2 (19%). 18:2/18:2 (15%), 18:1/18:1 (13%), 18:1/18:3 (8%), 16:0/18:1 (6%), 16:0/18:3 (6%) and 18:2/18:3 (2%).

### 3.2 Changes to molecular species during lipid accumulation

The development of an oil seed typically occurs in three main stages – cell division, oil accumulation and dehydration [58]. The rapid phase of oil accumulation in *B*. *napus* cv Westar is from around 20 DAF to 35 DAF [46], [47]. We analysed seeds from 20 DAF to represent early oil accumulation, 27 DAF as a mid-point and 35 DAF for towards the end of significant oil accumulation [46], [47].

**Fig. 3:**
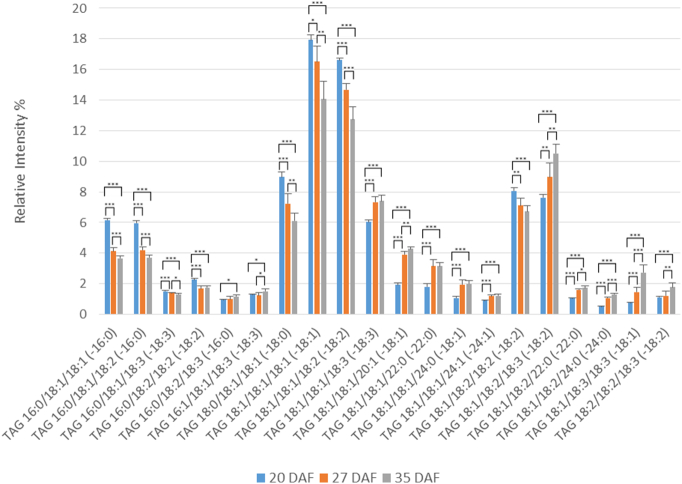
Changes in the percentages of major molecular species of triacylglycerol during oil accumulation in oilseed rape. Triacylglycerol molecular species were analysed by multiple reaction monitoring (MRM) using a triple quadrupole instrument after separation of lipid species by reverse phase liquid chromatography (RPLC) as described in Materials and methods. Three developmental time points were analysed, 20, 27 and 35 days after flowering (DAF), representing early, mid and late stages of oil accumulation respectively in *B*. *napus* cv. Westar. Means ± s.d. (*n* = 5) are shown. *P < 0.05, **P < 0.01, ***P < 0.001. Major molecular species (> 1% total) are detailed here, with further information on minor species listed in Supplementary Table 8. The amounts of triacylglycerol at the three stages were 15.17 ± 0.35, 72.55 ± 0.58 and 146.98 ± 0.84 μg fatty acid/mg FW, respectively, as measured by TLC/GLC.

Changes in the main TAG molecular species during the 20–35 DAF period are shown in Fig. 3. The most prominent species at all time points were 18:1/18:1/18:1 and 18:1/18:1/18:2, in keeping with the overall fatty acid composition of the accumulating TAG [2], [32]. The next most abundant TAG species were those containing a single saturated fatty acid (16:0/18:1/18:1, 16:0/18:1/18:2, 18:0/18:1/18:1) or more highly unsaturated species (18:1/18:1/18:3, 18:1/18:2/18:2, 18:1/18:2/18:3). The total detected TAG molecular species are given in Supplementary Table 8 where it will be noted that those TAG molecules containing VLCFAs are only minor components.

During the oil accumulation period there was a general shift towards more unsaturated TAG molecular species. This was particularly noticeable with those containing palmitate or stearate reducing between 20 and 27 DAF. In addition, although minor species, those with very VLCFAs tended to increase during the oil accumulation period (Supplementary Table 8).

**Fig. 4:**
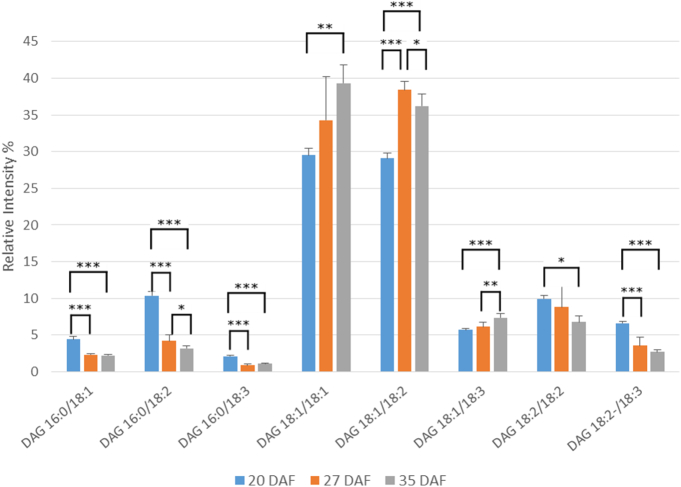
Changes in the percentages of major molecular species of diacylglycerol during oil accumulation in oilseed rape. Diacylglycerol molecular species were analysed by multiple reaction monitoring (MRM) using a triple quadrupole instrument after separation of lipid species by RPLC as described in Materials and methods. Three developmental time points were analysed, 20, 27 and 35 days after flowering (DAF), representing early, mid and late stages of oil accumulation, respectively, in *B*. *napus* cv. Westar. Means ± s.d. (*n* = 5) are shown. *P < 0.05, **P < 0.01, ***P < 0.001. Major molecular species (> 1% total) are detailed here, with further information on minor species listed in Supplementary Table 9. The amounts of diacylglycerol at the three stages were 0.23 ± 0.01, 0.66 ± 0.24 and 1.23 ± 0.14 μg fatty acid/mg FW, respectively, as measured by TLC/GLC.

The main molecular species of DAG at the three developmental time points are shown in Fig. 4. The major species at all times of oil accumulation were 18:1/18:1 and 18:1/18:2, each representing 30–40% of the total. Both of these species increase in abundance throughout development. Other significant molecular species of DAG were those containing palmitate (16:0/18:1, 16:0/18:2) or those which were more unsaturated (18:1/18:3, 18:2/18:2, 18:2/18:3). The palmitate-containing species as well as 18:2/18:3 decreased during oil accumulation (Fig. 4). There were only very small amounts of DAG molecular species (< 1%) containing palmitoleate or VLCFAs (Supplementary Table 9).

**Fig. 5:**
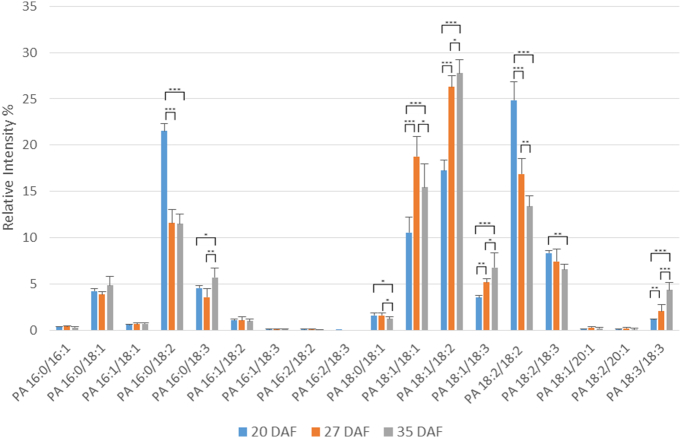
Changes in the percentages of major molecular species of phosphatidic acid during oil accumulation in oilseed rape. Phosphatidic acid molecular species were analysed by multiple reaction monitoring (MRM) using a triple quadrupole instrument after separation of lipid species by HILIC as described in Materials and methods. Three developmental time points were analysed, 20, 27 and 35 days after flowering (DAF), representing early, mid and late stages of oil accumulation, respectively, in *B*. *napus* cv. Westar. Means ± s.d. (*n* = 5) are shown. *P < 0.05, **P < 0.01, ***P < 0.001. Major molecular species (> 1% total) are detailed here, with further information on minor species listed in Supplementary Table 10. The amounts of phosphatidic acid at the three stages were 0.34 ± 0.25, 0.32 ± 0.14 and 0.11 ± 0.07 μg fatty acid/mg FW, respectively, as measured by TLC/GLC.

The main molecular species of PA, the immediate precursor to DAG in the Kennedy pathway, are shown in Fig. 5 and all detected species are given in Supplementary Table 10. The major species were 16:0/18:2, 18:1/18:1, 18:1/18:2 and 18:2/18:2. Between 20 and 27 DAF there were increases in the abundance of 18:1/18:1 and 18:1/18:2 species while 16:0/18:2 and 18:2/18:2 decreased (Fig. 5). Other significant PA species were 16:0/18:1, 16:0/18:3, 18:1/18:3, 18:2/18:3 and 18:3/18:3. In general, these maintained relatively steady proportions during oil accumulation.

**Fig. 6:**
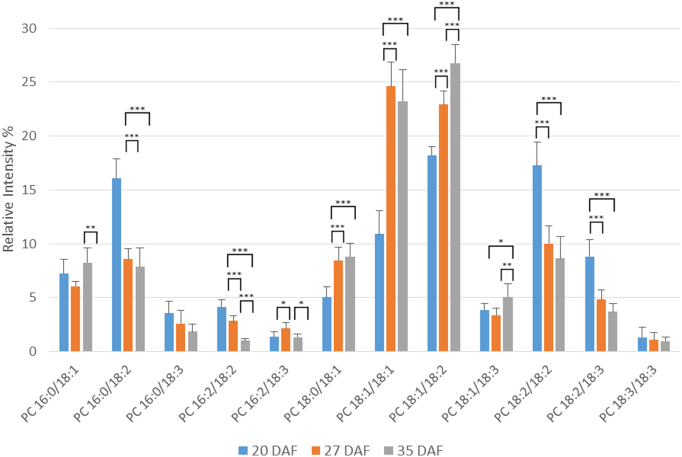
Changes in the percentages of major molecular species of phosphatidylcholine during oil accumulation in oilseed rape. Phosphatidylcholine molecular species were analysed by multiple reaction monitoring (MRM) using a triple quadrupole instrument after separation of the lipid classes by HILIC as described in Materials and methods. Three developmental time points were analysed, 20, 27 and 35 days after flowering (DAF), representing early, mid and late stages of oil accumulation, respectively, in *B*. *napus* cv. Westar. Means ± s.d. (*n* = 5) are shown. *P < 0.05, **P < 0.01, ***P < 0.001. Major molecular species (> 1% total) are detailed here, with further information on minor species listed in Supplementary Table 11. The amounts of phosphatidylcholine at the three stages were 1.37 ± 0.12, 2.37 ± 0.03 and 3.65 ± 0.08 μg fatty acid/mg FW, respectively, as measured by TLC/GLC.

PC plays an important role during oil accumulation in crops [1], [14]. The main molecular species of PC during oil accumulation in oilseed rape are shown in Fig. 6 and a complete analysis of all detected species in Supplementary Table 11. The two main molecular species at 27 and 35 DAF were 18:1/18:1 and 18:2/18:2 but both showed a significant rise compared to 20 DAF (Fig. 6). This increase in proportion was compensated by a decrease in the 16:0/18:2, 18:2/18:2 and 18:2/18:3 species by the mid stage of oil accumulation (i.e. between 20 and 27 DAF). Other significant PC molecular species (around 5%) were 18:0/18:1, which increased between 20 and 27 DAF and 16:0/18:1 and 18:1/18:3 which showed little change in proportion during development. There were only minor amounts of PC species containing palmitoleate or VLCFAs (Supplementary Table 11).

**Fig. 7:**
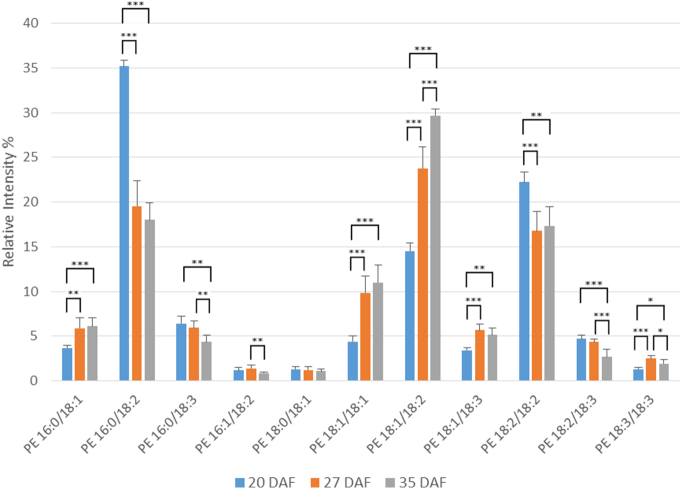
Changes in the percentages of major molecular species of phosphatidylethanolamine during oil accumulation in oilseed rape. Phosphatidylethanolamine molecular species were analysed by multiple reaction monitoring (MRM) using a triple quadrupole instrument after separation of the lipid classes by HILIC as described in Materials and methods. Three developmental time points were analysed, 20, 27 and 35 days after flowering (DAF), representing early, mid and late stages of oil accumulation, respectively, in *B*. *napus* cv. Westar. Means ± s.d. (*n* = 5) are shown. *P < 0.05, **P < 0.01, ***P < 0.001. Major molecular species (> 1% total) are detailed here, with further information on minor species listed in Supplementary Table 12. The amounts of phosphatidylethanolamine at the three stages were 0.42 ± 0.07, 0.38 ± 0.03 and 0.58 ± 0.04 μg fatty acid/mg FW, respectively, as measured by TLC/GLC.

PE is synthesised by a similar pathway to PC [55], [58], using DAG from the Kennedy pathway. However, unlike PC, it does not appear to be involved in active participation in seed oil accumulation [14], [15], [57] nor as a significant substrate for fatty acid desaturation reactions [56], [59]. Therefore, we examined PE molecular species to compare with those of PC. The main molecular species of PE are shown in Fig. 7 with a complete breakdown of all species detected in Supplementary Table 12. The pattern of molecular species was noticeably different from those of PC (Fig. 6). While 18:1/18:2 was a major species, so were 18:2/18:2 and 16:0/18:2 (Fig. 7). The latter was the main species at 20 DAF. 16:0/18:1, 18:1/18:1, 18:1/18:2 and 18:1/18:3 all showed increases between 20 and 27 DAF while 16:0/18:2 decreased. Other significant species, each of which showed little change in proportion during oil accumulation, were 16:0/18:3, 18:2/18:3 and 18:3/18:3.

In summary, PE, despite being formed by the same Kennedy pathway as PC, showed very distinct differences in its molecular species proportions, in keeping with the important role of PC (but not PE) in fatty acid desaturation and in TAG accumulation.

### 3.3 Measurement of the acyl-CoA pool during seed development

**Fig. 8:**
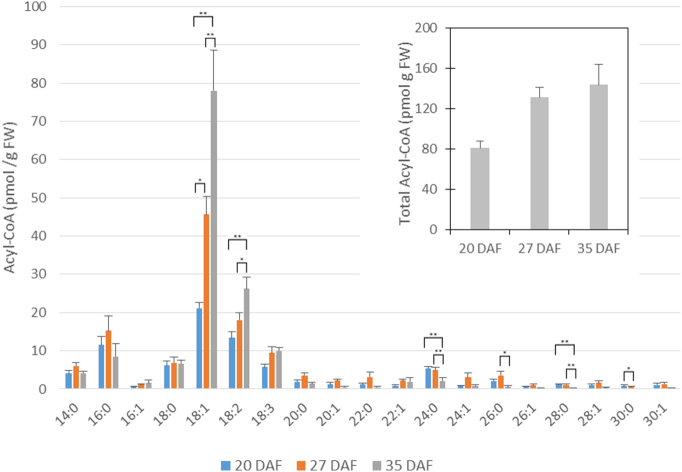
Distribution of acyl-CoA species during the rapid period of oil accumulation in oilseed rape. Means ± S.D. (*n* = 5). *P < 0.05, **P < 0.01, ***P < 0.001. The inset shows the total amount of acyl-CoAs at the three development times.

A diverse variety of acyl-CoAs were detected in the developing oilseed rape seeds ranging from the medium-chain myristoyl-CoA up to very long chains of thirty carbons (Fig. 8). However, as expected, the main acyl-CoAs present at all developmental points analysed (20–35 DAF) were oleoyl-, linoleoyl-, palmitoyl-, linolenoyl- and stearoyl- (in order of abundance). During oil accumulation, the amounts of oleoyl-CoA, linoleoyl-CoA and linolenoyl-CoA increased significantly. Because these were major components of the acyl-CoA pool, the total level almost doubled from 20 to 35 DAF. There were a large number of very long chain acyl-CoAs. In general, these were both saturated and the n-9 monoene equivalents. The very long chain acyl-CoAs were most abundant at the intermediate oil accumulation time point (27 DAF) and reduced markedly by the latest (35 DAF) stage.

### 3.4 Can lipidomics inform judgements about enzymes used for triacylglycerol accumulation?

It is accepted that there are several enzymes that can contribute to TAG accumulation in oil crops, apart from the direct Kennedy pathway. These particularly involve PDAT and metabolism around PC [1]. Direct measurement of enzyme activities, such as PDAT and DGAT, can provide some information [12] but, of course, these are measured in vitro under optimised conditions. Gene deletion is another technique and has been applied to the analogous plant arabidopsis [37], but again, there are problems for quantitative interpretation.

Thus, apart from the important information supplied by the lipidomic data per se, we sought to use them to evaluate the possible contributions of DGAT and PDAT towards TAG accumulation. We chose to use the mid-point of oil accumulation (27 DAF) for the evaluation since this was typical of high biosynthetic rates and was not unduly influenced by those tissues which were not primarily oleaginous. It was clear from the acyl-CoA data (Fig. 8) that there was selectivity in the fatty acids used for TAG formation with, for example, a low incorporation of myristate (e.g. Fig. 1 and Supplementary Table 4). We used the acyl-CoA data from Fig. 8 for 27 DAF as potential substrates for DGAT. For PDAT we used the data for PC (Fig. 6) and assumed an enrichment at the sn-2 position of unsaturated fatty acids. Although we have no data for *B*. *napus* cv. Westar, positional analysis of PC from a variety of plants [60], [61], [62] suggests that the distribution of fatty acids in the major molecular species (see Supplementary Table 11) would be 6% 16:0, 9% 18:0, 54% 18:1, 65% 18:2, 70% 18:3 for each fatty acid respectively, at the sn-2 position. The only VLCFA detected in PC was 20:1, found in two very minor species, 18:1/20:1 and 18:2/20:1. We have used the above crude approximation of sn-2 distributions in our calculations. Moreover, although there is no data specifically for oilseed rape, PDAT is assumed to use fatty acids from the sn-2 position of PC [16]. In addition, our data represent a steady-state profile of seed lipids and, since there is constant re-modelling of these, such metabolism may mask accurate evaluation of DGAT and PDAT.

In Table 1 we have a comparison of the molecular species composition of TAG formed exclusively by the Kennedy pathway (DGAT) or exclusively via PDAT with the actual data for accumulated TAG at 27 DAF (the data for DGAT and for PDAT assume no substrate selectivity). It will be clear that while neither pathway gives a good match, the molecular species of TAG that accumulated were nearer to those predicted from calculation of DGAT activity rather than PDAT. This would be in keeping with in vitro estimation of activity [12] even though we concur with the view that PDAT is likely to have significant input in vivo, as suggested originally from gene suppression experiments with arabidopsis [37].

**Table 1:** Comparison of the distribution of TAG molecular species if they were generated by DGAT or PDAT only with the actual percentages found. For the assumptions made in the calculations, see the text in Section 3.4.

Molecular species	% Molecular species		
	PDAT	DGAT	Actual TAG
16:0/18:1/18:1	1	7	6
16:0/18:1/18:2	2	8	5
16:0/18:2/18:2	2	2	2
16:0/18:1/18:3	< 1	1	1
16:0/18:2/18:3	< 1	< 2	1
18:0/18:1/18:1	–	2	7
18:1/18:1/18:1	20	17	20
18:1/18:1/18:2	35	26	16
18:1/18:1/18:3	5	7	7
18:1/18:1/20:0	–	1	3
18:1/18:1/20:1	< 1	–	4
18:1/18:1/24:0	–	1	2
18:1/18:1/24:1	–	1	1
18:1/18:2/18:2	20	11	6
18:1/18:2/18:3	6	7	8
18:1/18:2/22:0	–	1	2
18:1/18:3/18:3	1	1	2
18:2/18:2/18:3	2	2	1

Nevertheless, there are several TAG molecular species whose percentages are significantly different from calculated amounts. 18:0/18:1/18:1 and all species containing VLCFAs (20-24C) are higher (Supplementary Tables 4 and 8) than calculated for the Kennedy (DGAT) pathway while 18:1/18:1/18:2 and 18:1/18:2/18:2 are lower. Clearly, substrate selectivity, for which we have little information, must play an important role.

It is also worth noting that, although generally DGAT can account for those species containing VLCFs much better than PDAT (Table 1), the acyl-CoA pool contained much lower amounts of 20:1 than several other very long chain monoenes (Supplementary Table 13). Therefore, because DAG contained no detectable 20:1 (Supplementary Tables 1 and 7) there must have been strong substrate selectivity for erucoyl-CoA by the DGAT enzyme.

## 4 Discussion

With recent discoveries of new enzymes involved in lipid accumulation in oil crops, it is clear that our understanding of metabolism and particularly its regulation, is incomplete [1], [4], [6], [9], [14], [63]. We have been using a number of techniques to reveal features of the control of TAG biosynthesis in a major oil crop, *B*. *napus*. These include classic biochemistry [33], [34], genetic manipulation [35], flux control experiments [12] and MALDI-MSI to examine spatial and temporal aspects [46]. Here we report detailed lipidomic examination of important lipid classes during the rapid period of lipid accumulation in oilseed rape.

Analysis of seeds at the mid-point of oil accumulation (27 DAF) revealed the major molecular species detectable and confirmed robustness of the measurements. When comparing the non-polar lipids, DAG and TAG (Fig. 1), the major DAG species (18:1/18:1 and 18:1/18:2) clearly accounted for major TAG species. However, TAG contained significant amounts of stearate as well as VLCFAs (Fig. 1 and Suppl. Table 4) which were not detected in DAG (Fig. 1 and Suppl. Table 3). Although the VLCFAs are all found in the acyl-CoA pool at 27 DAF (Fig. 8), they were not detected in PC at this time (Fig. 2 and Suppl. Tables 6 and 11). This suggests that TAG species containing VLCFAs originate from the DGAT reaction (see also Section 3.4).

For seeds aged 27 DAF, the pattern of molecular species in PA was distinct from that of DAG, even though they are directly connected in the Kennedy pathway for TAG formulation. This, presumably, reflects the very significant flux of carbon between DAG and PC [1], [4], [14] and the activity of FAD2 and FAD3 which use PC as substrate [56]. In addition, PA is used for synthesis of anionic phosphoglycerides [24], [55], [58], however such reactions are likely to be minor in maturing oilseed rape seeds. Indeed, radiolabelling of *B*. *napus* seeds with either [1-^14^C] acetate or [U^14^—C] glycerol gave only very minor incorporation into cardiolipin (diphosphatidylglycerol), PG or PI (H. Woodfield, unpublished data).

Since both PC and PE are formed by a CDP-base pathway using DAG [24], [55], [58] (PE can also be converted to PC by methylation [58], [64]) we included an examination of PE (as the second major phospholipid) in our study. Fig. 2 shows that the distribution of molecular species in PC and PE at 27 DAF is clearly different. This is to be expected from the intimate involvement of PC, but not PE in TAG synthesis [1], [4], [14]. In addition, evidence has suggested the cholinephosphotransferase and ethanolaminephosphotransferase enzymes (catalysing the final step in the CDP-base pathway) have been shown to have distinct characteristics in soybean [65], [66], [67], although an isolated gene codes for both activities [68]. On the other hand, two yeast enzymes which both show equal similarity to the deduced amino acid sequence of the soybean gene [68] have distinct characteristics [69].

For the rapid period of oil accumulation, we used samples of 20–35 DAF which we determined were appropriate in the cultivar used and in agreement with other studies of *B*. *napus* [38], [47]. Although the major species of TAG and DAG remained broadly constant, there were significant changes in their percentage distribution from early to late oil accumulation (Fig. 3, Fig. 4). For TAG there was a general increase in more unsaturated species and in those containing VLCFAs during oil accumulation (Fig. 3 and Suppl. Table 8). This was not reflected in the DAG species (Fig. 4 and Suppl. Table 9), indicating the substrate selectivity of DGAT [63] and/or the presence of separate pools of DAG [4], [8], [14]. As discussed above, the absence of significant VLCFAs in PC, preclude a role for PDAT in giving rise to the differences in VLCFA content between DAG and TAG species with seed maturation. In contrast to the non-polar lipids, all three of the phosphoglycerides showed significant changes in the relative distribution of their molecular species during oil accumulation. Although PA, PC and PE each had a distinct distribution of molecular species, there were some consistent changes in major species with maturation. Thus, all three phosphoglycerides showed decreases in 16:0/18:2 and increases in 18:1/18:1 and 18:1/18:2, particularly in the period 20–27 DAF. These data corroborated previous measurements made via MALDI-MS [46].

As remarked before, PA, PC and PE all had distinct differences in their distribution of molecular species, despite their metabolic connections [55], [58]. There were also differences in the changes to their molecular species during maturation. For example, while PA and PC both showed a large decrease in their 18:2/18:2 species during oil accumulation (Fig. 5, Fig. 6), PE did not (Fig. 7) similarly, there were differences in the changes in species such as 18:0/18:1, 18:1/18:3 and 18:3/18:3. All this points to the subtle distinctions in metabolism for phosphoglycerides which undoubtable reflects their different functions in plant cells [70].

Although there are significant differences in the molecular species of PC and PE, and indeed in their proportional changes in amounts during maturation (Fig. 6, Fig. 7), the major species alter in the same direction. For instance, 16:0/18:2 and 18:2/18:2 both decrease while 18:1/18:1 and 18:1/18:2 increase. These changes are likely to reflect the parallel alterations in DAG species (Fig. 4), since the latter is the common substrate for PC and PE formation by the Kennedy pathway [24].

A key question that has exercised plant scientists since the discovery of PDAT [16] has been its contribution to TAG synthesis relative to DGAT in different plants. This is important for efforts to increase oil production since, although over-expression of DGAT seems to be effective in increasing TAG accumulation either alone [35], [36] or as part of a ‘push/pull’ strategy [28], [29], information about PDAT is less clear and suggests that it is less important for crops producing high oleate such as oilseed rape [14]. Indeed, overexpression or knockout of Arabidopsis PDAT1 resulted in significant changes in oil content (and fatty acid composition) in leaves but not in seeds [71]. Nevertheless, it has been shown that DGAT and PDAT have overlapping function for oil accumulation in plants [9], [37] and, depending on the crops, their relative contribution may be quite different [4], [62], [72]. Evidence is accumulating that PDAT may be particularly important for incorporation of unusual (e.g. hydroxyl) fatty acids [1], [4] and, to a certain extent, for highly unsaturated molecules [63].

A recent paper has addressed such questions in *Camelina sativa* where it was concluded that TAG accumulation was dominated by DGAT in the cotyledon tissues but, in the plant, PDAT could compensate for an absence of DGAT with no reduction in seed oil content [73].

Efforts to address the question of the relative importance of DGAT and PDAT for TAG synthesis in oil crops have included measurements of enzyme activity in vitro [10], [11], [12], [63] and genomic manipulation [37], [71]. Even when measurements in vitro have been carried out very carefully and in detail [63] there is always the problem that enzyme specificity determinations in vitro may not reflect the selectivity (and, hence, activity) at the substrate concentrations in vivo. Another method that has been applied to soybean in particular in to use radiolabelling from different precursors [14].

Although previous studies with in vitro enzyme measurements [12] and transcription levels [38] suggested that DGAT was more important for overall flux into TAG in oilseed rape, we were interested to see whether our molecular species measurements could add useful information. This was particularly important in view of the current uses of genetic manipulation to enhance oil production [6], [72], [74], [75], [76]. Indeed, Bates has recently commented that the relative importance of DGAT versus PDAT is a major uncertainty in plant lipid metabolism [14].

As discussed in Section 3.4 we had to make assumptions in order to calculate how the activities of DGAT or PDAT could account for the final molecular species in TAG. These assumptions included the availability of all acyl-CoAs in the proportions detected (Fig. 8), an enrichment of unsaturated fatty acids at the sn-2 position of PC [60], [61], [62] and utilisation of fatty acids at the sn-2 position by PDAT [16]. For the latter, recent experiments for safflower and sunflower suggest that fatty acids at the sn-1 position can also be used (at a quarter of the rate for the sn-2 position) [63]. Two main conclusions can be made, first, the pattern of TAG species matches better to an exclusive formation by DGAT than by PDAT. Second, the actual TAG species distribution shows large differences from calculated patterns, especially for 18:0/18:1/18:1, 18:1/18:1/18:2 and 18:1/18:2/18:2 (Table 1).

As the apparent preferred use of DGAT over PDAT matches measurement of these enzyme activities in vitro [12] and also the transcriptome analysis based on EST sequencing where the PDAT orthologs of *B*. *napus* were much lower than DGAT ESTs [38]. For *B*. *napus* seeds the DGAT1 has much higher activity than DGAT2 [35] and over-expression of this isoform was effective in increasing oil yields in greenhouse experiments and in field trials [36]. Again, this agreed with measurements of ESTs in *B*. *napus* [38]. The differences noted in Table 1 are obviously due in a major part to substrate selectivity. Unfortunately, we have no information for the oilseed rape enzymes but experiments in other plant tissues revealed substrate specificity for PDAT [63] and, especially, for DGAT [57], [63], [77]. Further experiments in this area, especially for major oil crops, would be timely.

## 5 Conclusions

The data reported here describe lipidomic analysis of major lipids involved in TAG formation in the major oil crop, *B*. *napus*. They are one of the first such analyses of a developing plant tissue – the maturing oilseed. The results show quite distinct molecular species distributions in the various lipid classes and demonstrate the complex nature of metabolism during oil accumulation. Theoretical calculations to compare the possible selective contributions of DGAT or PDAT during TAG biosynthesis suggest that DGAT is more important. However, the actual pattern of TAG molecular species shows clearly that enzyme selectivity has major importance in forming the accumulating oil. Our experiments contribute significantly towards understanding how the final storage lipid is formed within the world's third most important oil crop.

The following are the supplementary data related to this article.

Supplementary Table 1 MS/MS data for TAG and DAG. Supplementary Table 1

Supplementary Table 2 MRM list for phosphoglycerides. Supplementary Table 2

Supplementary tables Image 1

## Transparency document

Transparency document. Image 2
