# Supplementary material for: Using lipidomics to reveal details of lipid accumulation in developing seeds from oilseed rape (Brassica napus L.)
Source: Biochim Biophys Acta. 2018 Mar;1863(3):339–48. doi: 10.1016/j.bbalip.2017.12.010 (PMC5791847; doi:10.1016/j.bbalip.2017.12.010)
Supplement: Supplementary file 3 — Supplementary tables [file mmc3.pdf]

Supplementary Table 1. MS/MS data for TAG and DAG

See attached supplementary Excel file

Supplementary Table 2. MRM list for phosphoglycerides

See attached supplementary Excel file

Supplementary Table 3. Molecular species of diacylglycerol (DAG) from seeds of 27 DAF. Means ± s.d. (n=5).

Molecular species	Sample A	Sample B	Sample C	Sample D
**DAG 34:1 (16:0/18:1)**	3.2 ± 0.1	2.8 ± 0.1	3.2 ± 0.0	2.9 ± 0.1
**DAG 34:2 (16:0/18:2)**	3.3 ± 0.2	3.1 ± 0.1	3.6 ± 0.2	3.3 ± 0.2
**DAG 34:2 (16:1/18:1)**	0.5 ± 0.0	0.3 ± 0.0	0.4 ± 0.0	0.4 ± 0.0
**DAG 36:2 (18:1/18:1)**	46.5 ± 1.2	44.7 ± 0.5	43.7 ± 0.1	45.9 ± 0.4
**DAG 36:3 (18:1/18:2)**	31.7 ± 0.6	34.0 ± 0.2	30.9 ± 0.6	33.0 ± 0.0
**DAG 36:4 (18:2/18:2)**	4.8 ± 0.1	5.3 ± 0.3	5.0 ± 0.3	5.1 ± 0.2
**DAG 36:4 (18:1/18:3)**	6.6 ± 0.2	6.7 ± 0.1	9.5 ± 0.1	6.5 ± 0.1
**DAG 36:5 (18:2/18:3)**	3.3 ± 0.2	3.2 ± 0.1	3.5 ± 0.1	2.9 ± 0.1

Supplementary Table 4. Molecular species of triacylglycerol (TAG) from seeds of 27 DAF. Means ± s.d. (n=5).

	Sample A			Sample B			Sample C			Sample D		
**TAG 14:0/18:1/18:1 (-14:0)**	0.27	±	0.01	0.27	±	0.01	0.21	±	0.00	0.27	±	0.01
**TAG 14:0/18:1/18:2 (-14:0)**	0.18	±	0.00	0.19	±	0.01	0.17	±	0.00	0.20	±	0.00
**TAG 16:0/16:0/18:1 (-18:1)**	0.35	±	0.01	0.33	±	0.00	0.39	±	0.02	0.33	±	0.01
**TAG 16:0/16:0/18:2 (-18:2)**	0.30	±	0.02	0.28	±	0.07	0.44	±	0.00	0.34	±	0.00
**TAG 16:0/16:1/18:1 (-16:1)**	0.19	±	0.01	0.17	±	0.00	0.19	±	0.00	0.18	±	0.00
**TAG 16:0/16:1/18:2 (-16:0)**	0.38	±	0.01	0.38	±	0.01	0.49	±	0.01	0.38	±	0.01
**TAG 16:0/18:1/18:1 (-16:0)**	5.75	±	0.12	5.50	±	0.06	6.15	±	0.13	5.58	±	0.06
**TAG 16:0/18:1/18:2 (-16:0)**	4.58	±	0.02	4.69	±	0.05	5.77	±	0.04	4.71	±	0.03
**TAG 16:0/18:1/18:3 (-18:3)**	1.28	±	0.02	1.29	±	0.03	1.66	±	0.02	1.27	±	0.04
**TAG 16:0/18:1/24:0 (-24:0)**	0.25	±	0.02	0.20	±	0.01	0.15	±	0.01	0.23	±	0.01
**TAG 16:0/18:2/18:2 (-18:2)**	1.08	±	0.02	1.15	±	0.01	1.40	±	0.02	1.14	±	0.02
**TAG 16:0/18:2/18:3 (-16:0)**	0.72	±	0.02	0.83	±	0.02	1.14	±	0.00	0.79	±	0.01
**TAG 16:0/18:3/18:3 (-16:0)**	0.13	±	0.00	0.16	±	0.01	0.24	±	0.01	0.14	±	0.00
**TAG 16:1/18:1/18:3 (-18:3)**	0.77	±	0.02	0.85	±	0.02	1.09	±	0.01	0.81	±	0.03
**TAG 16:1/18:2/18:2 (-16:1)**	0.36	±	0.00	0.30	±	0.01	0.33	±	0.00	0.36	±	0.02
**TAG 16:1/18:2/18:3 (-16:1)**	0.08	±	0.01	0.07	±	0.00	0.08	±	0.00	0.08	±	0.00
**TAG 16:2/18:1/18:2 (-16:2)**	0.14	±	0.01	0.13	±	0.00	0.10	±	0.00	0.14	±	0.01
**TAG 16:2/18:1/18:3 (-18:3)**	0.31	±	0.01	0.33	±	0.01	0.45	±	0.03	0.31	±	0.00
**TAG 16:2/18:2/18:2 (-16:2)**	0.06	±	0.01	0.06	±	0.00	0.05	±	0.00	0.06	±	0.00
**TAG 16:3/18:1/18:1 (-16:3)**	0.29	±	0.01	0.27	±	0.01	0.18	±	0.00	0.27	±	0.01
**TAG 16:3/18:2/18:1 (-16:3)**	0.13	±	0.01	0.13	±	0.00	0.09	±	0.01	0.13	±	0.01
**TAG 18:0/18:1/18:1 (-18:0)**	8.43	±	0.02	7.91	±	0.11	6.19	±	0.06	7.21	±	0.09
**TAG 18:0/18:1/20:1 (-20:1)**	0.34	±	0.01	0.32	±	0.00	0.22	±	0.01	0.30	±	0.00
**TAG 18:1/18:1/18:1 (-18:1)**	22.57	±	0.35	21.51	±	0.51	20.48	±	0.21	21.92	±	0.22
**TAG 18:1/18:1/18:2 (-18:2)**	15.88	±	0.09	16.10	±	0.13	15.64	±	0.07	16.25	±	0.09
**TAG 18:1/18:1/18:3 (-18:3)**	7.24	±	0.03	7.10	±	0.03	7.68	±	0.13	7.25	±	0.06
**TAG 18:1/18:1/20:1 (-18:1)**	4.14	±	0.05	3.97	±	0.07	3.26	±	0.03	4.19	±	0.06
**TAG 18:1/18:1/22:0 (-22:0)**	2.32	±	0.06	2.25	±	0.06	1.54	±	0.06	2.15	±	0.03
**TAG 18:1/18:1/24:0 (-18:1)**	1.59	±	0.06	1.29	±	0.07	0.81	±	0.06	1.42	±	0.06
**TAG 18:1/18:2/24:0 (-24:0)**	0.94	±	0.02	0.84	±	0.00	0.79	±	0.02	0.98	±	0.04
**TAG 18:1/18:1/26:1 (-18:1)**	0.19	±	0.01	0.14	±	0.01	0.11	±	0.00	0.16	±	0.01
**TAG 18:1/18:2/18:2 (-18:2)**	5.00	±	0.08	5.58	±	0.03	5.67	±	0.06	5.49	±	0.04
**TAG 18:1/18:2/18:3 (-18:2)**	6.35	±	0.10	7.40	±	0.14	7.87	±	0.10	7.03	±	0.10
**TAG 18:1/18:2/20:1 (-20:1)**	0.86	±	0.01	0.93	±	0.02	1.04	±	0.02	0.96	±	0.01
**TAG 18:1/18:2/22:0 (-22:0)**	1.15	±	0.02	1.20	±	0.04	1.14	±	0.05	1.19	±	0.04
**TAG 18:1/18:1/24:1(-24:1)**	0.77	±	0.02	0.73	±	0.04	0.61	±	0.01	0.79	±	0.02
**TAG 18:1/18:2/24:1 (-24:1)**	0.35	±	0.00	0.39	±	0.01	0.41	±	0.01	0.40	±	0.02
**TAG 18:1/18:3/18:3 (-18:1)**	1.19	±	0.03	1.39	±	0.05	1.85	±	0.04	1.31	±	0.03
**TAG 18:1/18:3/20:1 (-18:3)**	0.39	±	0.01	0.38	±	0.01	0.48	±	0.01	0.39	±	0.00
**TAG 18:1/18:3/22:0 (-18:3)**	0.27	±	0.01	0.28	±	0.01	0.32	±	0.01	0.29	±	0.01
**TAG 18:2/18:2/18:3 (-18:2)**	0.85	±	0.05	1.03	±	0.08	1.11	±	0.03	0.96	±	0.02
**TAG 18:2/18:2/20:0 (-20:0)**	0.60	±	0.01	0.62	±	0.01	0.81	±	0.01	0.58	±	0.01
**TAG 18:2/18:2/20:1 (-18:2)**	0.17	±	0.00	0.18	±	0.00	0.22	±	0.00	0.18	±	0.00
**TAG 18:2/18:2/22:0 (-18:2)**	0.17	±	0.01	0.19	±	0.01	0.22	±	0.01	0.19	±	0.01
**TAG 18:2/18:2/24:0 (-24:0)**	0.38	±	0.01	0.35	±	0.01	0.38	±	0.01	0.39	±	0.01
**TAG 18:2/18:3/18:3 (-18:2)**	0.28	±	0.02	0.31	±	0.02	0.37	±	0.02	0.30	±	0.02

Supplementary Table 5. Molecular species of phosphatidic acid (PA) from seeds of 27 DAF. Means ± s.d. (n=5).

	Sample A	Sample B	Sample C	Sample D
**PA 16:0/18:2**	13.1 ± 0.7	13.0 ± 1.3	14.8 ± 1.4	14.7 ± 0.6
**PA 16:0/18:3**	4.5 ± 0.6	4.2 ± 0.2	4.9 ± 0.2	5.1 ± 0.3
**PA 16:1/18:1**	0.8 ± 0.1	0.8 ± 0.1	0.8 ± 0.2	0.6 ± 0.1
**PA 18:1/18:1**	31.5 ± 1.1	30.1 ± 0.8	28.5 ± 1.3	29.8 ± 1.0
**PA 18:1/18:2**	27.4 ± 0.6	30.0 ± 0.9	25.9 ± 0.9	25.2 ± 0.8
**PA 18:2/18:2**	14.7 ± 0.3	13.9 ± 1.6	16.1 ± 0.2	15.5 ± 0.2
**PA 18:2/18:3**	8.0 ± 0.3	7.9 ± 0.3	9.1 ± 0.6	9.2 ± 0.8

Supplementary Table 6. Molecular species of phosphatidylcholine (PC) from seeds of 27 DAF. Means ± s.d. (n=5).

	Sample A	Sample B	Sample C	Sample D
**PC 16:0/18:1**	9.3 ± 0.5	8.9 ± 0.9	9.5 ± 0.2	9.7 ± 0.5
**PC 16:0/18:2**	7.2 ± 0.1	7.7 ± 0.1	9.2 ± 0.1	7.5 ± 0.4
**PC 16:0/18:3**	2.4 ± 0.2	2.4 ± 0.3	2.7 ± 0.2	2.3 ± 0.2
**PC 18:1/18:1**	40.6 ± 0.9	36.5 ± 0.7	36.3 ± 0.7	38.8 ± 0.2
**PC 18:1/18:2**	26.1 ± 0.5	28.7 ± 0.8	24.8 ± 0.9	27.4 ± 0.3
**PC 18:1/18:3**	6.2 ± 0.7	6.2 ± 0.4	8.3 ± 0.3	6.1 ± 0.1
**PC 18:2/18:2**	7.2 ± 0.5	8.6 ± 0.2	7.9 ± 0.5	7.4 ± 0.4
**PC 18:3/18:3**	0.9 ± 0.1	0.9 ± 0.1	1.4 ± 0.1	1.0 ± 0.0

Supplementary Table 7. Molecular species of phosphatidylethanolamine (PE) from seeds of 27 DAF. Means ± s.d. (n=5).

	Sample A			Sample B			Sample C			Sample D		
**PE 16:0/18:1**	6.68	±	0.29	5.91	±	0.66	5.54	±	0.36	6.74	±	0.33
**PE 16:0/18:2**	17.96	±	0.30	19.18	±	0.26	20.97	±	0.15	18.57	±	0.23
**PE 16:0/18:3**	4.99	±	0.04	5.54	±	0.24	6.38	±	0.18	5.23	±	0.18
**PE 16:1/18:1**	0.46	±	0.04	0.52	±	0.01	0.52	±	0.07	0.42	±	0.02
**PE 16:1/18:2**	0.84	±	0.05	0.86	±	0.05	1.07	±	0.10	0.97	±	0.04
**PE 18:0/18:1**	1.79	±	0.12	1.30	±	0.15	1.31	±	0.07	1.31	±	0.10
**PE 18:1/18:1**	14.70	±	0.47	11.92	±	0.18	11.18	±	0.43	13.23	±	0.26
**PE 18:1/18:2**	24.71	±	0.42	24.37	±	0.48	21.42	±	0.77	24.99	±	0.13
**PE 18:1/18:3**	7.65	±	0.58	7.85	±	0.19	9.33	±	0.49	7.62	±	0.09
**PE 18:2/18:2**	14.78	±	0.22	16.16	±	0.55	15.31	±	0.33	15.29	±	0.37
**PE 18:2/18:3**	3.77	±	0.25	4.35	±	0.21	4.34	±	0.11	3.94	±	0.18
**PE 18:3/18:3**	1.66	±	0.13	2.03	±	0.10	2.63	±	0.10	1.69	±	0.19

Supplementary Table 8. Changes in molecular species of triacylglycerol (TAG) during seed development. Means ± s.d. (n=5).

	20 DAF			27 DAF			35 DAF		
**TAG 14:0/18:1/18:1 (-14:0)**	0.2	±	0.0	0.2	±	0.0	0.2	±	0.0
**TAG 14:0/18:1/18:2 (-14:0)**	0.2	±	0.0	0.2	±	0.0	0.3	±	0.0
**TAG 16:0/16:0/18:1 (-18:1)**	0.4	±	0.0	0.2	±	0.0	0.2	±	0.0
**TAG 16:0/16:0/18:2 (-18:2)**	0.5	±	0.0	0.3	±	0.0	0.3	±	0.0
**TAG 16:0/16:1/18:1 (-16:1)**	0.4	±	0.0	0.2	±	0.0	0.2	±	0.0
**TAG 16:0/16:1/18:2 (-16:0)**	0.5	±	0.0	0.4	±	0.0	0.5	±	0.0
**TAG 16:0/18:1/18:1 (-16:0)**	6.2	±	0.1	4.1	±	0.2	3.7	±	0.2
**TAG 16:0/18:1/18:2 (-16:0)**	6.0	±	0.2	4.2	±	0.2	3.7	±	0.1
**TAG 16:0/18:1/18:3 (-18:3)**	1.5	±	0.0	1.4	±	0.0	1.3	±	0.0
**TAG 16:0/18:1/24:0 (-24:0)**	0.2	±	0.0	0.2	±	0.0	0.2	±	0.0
**TAG 16:0/18:2/18:2 (-18:2)**	2.3	±	0.1	1.7	±	0.2	1.8	±	0.1
**TAG 16:0/18:2/18:3 (-16:0)**	0.9	±	0.1	1.0	±	0.2	1.2	±	0.1
**TAG 16:0/18:3/18:3 (-16:0)**	0.1	±	0.0	0.2	±	0.0	0.3	±	0.0
**TAG 16:1/18:1/18:3 (-18:3)**	1.3	±	0.1	1.2	±	0.2	1.5	±	0.2
**TAG 16:1/18:2/18:2 (-16:1)**	0.7	±	0.0	0.6	±	0.0	0.8	±	0.1
**TAG 16:1/18:2/18:3 (-16:1)**	0.1	±	0.0	0.1	±	0.0	0.2	±	0.0
**TAG 16:2/18:1/18:2 (-16:2)**	0.1	±	0.0	0.2	±	0.0	0.3	±	0.0
**TAG 16:2/18:1/18:3 (-18:3)**	0.3	±	0.0	0.5	±	0.1	0.8	±	0.1
**TAG 16:2/18:2/18:2 (-16:2)**	0.0	±	0.0	0.1	±	0.0	0.2	±	0.0
**TAG 16:3/18:1/18:1 (-16:3)**	0.2	±	0.0	0.5	±	0.1	0.6	±	0.0
**TAG 16:3/18:2/18:1 (-16:3)**	0.1	±	0.0	0.2	±	0.0	0.5	±	0.1
**TAG 18:0/18:1/18:1 (-18:0)**	9.0	±	0.3	7.2	±	0.6	6.1	±	0.5
**TAG 18:0/18:1/20:1 (-20:1)**	0.2	±	0.0	0.4	±	0.0	0.4	±	0.0
**TAG 18:1/18:1/18:1 (-18:1)**	17.9	±	0.3	16.5	±	1.0	14.1	±	1.1
**TAG 18:1/18:1/18:2 (-18:2)**	16.6	±	0.1	14.7	±	0.4	12.7	±	0.8
**TAG 18:1/18:1/18:3 (-18:3)**	6.0	±	0.2	7.3	±	0.4	7.4	±	0.4
**TAG 18:1/18:1/20:1 (-18:1)**	1.9	±	0.1	3.9	±	0.2	4.3	±	0.1
**TAG 18:1/18:1/22:0 (-22:0)**	1.8	±	0.2	3.2	±	0.4	3.2	±	0.2
**TAG 18:1/18:1/24:0 (-18:1)**	1.1	±	0.1	1.9	±	0.3	2.0	±	0.2
**TAG 18:1/18:1/24:0 (-24:0)**	0.9	±	0.0	1.2	±	0.1	1.2	±	0.1
**TAG 18:1/18:1/26:1 (-18:1)**	0.3	±	0.0	0.3	±	0.0	0.3	±	0.0
**TAG 18:1/18:2/18:2 (-18:2)**	8.1	±	0.2	7.1	±	0.5	6.7	±	0.3
**TAG 18:1/18:2/18:3 (-18:2)**	7.6	±	0.2	9.0	±	0.9	10.5	±	0.6
**TAG 18:1/18:2/20:1 (-20:1)**	0.5	±	0.0	0.8	±	0.1	0.9	±	0.1
**TAG 18:1/18:2/22:0 (-22:0)**	1.0	±	0.0	1.6	±	0.1	1.7	±	0.1
**TAG 18:1/18:2/24:1 (-24:1)**	0.5	±	0.0	1.1	±	0.1	1.3	±	0.1
**TAG 18:1/18:2/24:1 (-24:1)**	0.3	±	0.0	0.5	±	0.1	0.7	±	0.1
**TAG 18:1/18:3/18:3 (-18:1)**	0.8	±	0.0	1.5	±	0.3	2.7	±	0.5
**TAG 18:1/18:3/20:1 (-18:3)**	0.2	±	0.0	0.4	±	0.0	0.5	±	0.0
**TAG 18:1/18:3/22:0 (-18:3)**	0.2	±	0.0	0.4	±	0.0	0.5	±	0.0
**TAG 18:2/18:2/18:3 (-18:2)**	1.1	±	0.1	1.2	±	0.3	1.8	±	0.3
**TAG 18:2/18:2/20:0 (-20:0)**	0.9	±	0.1	0.8	±	0.1	0.7	±	0.1
**TAG 18:2/18:2/20:1 (-18:2)**	0.2	±	0.0	0.2	±	0.0	0.2	±	0.0
**TAG 18:2/18:2/22:0 (-18:2)**	0.2	±	0.0	0.3	±	0.0	0.3	±	0.1
**TAG 18:2/18:2/24:0 (-24:0)**	0.3	±	0.0	0.5	±	0.0	0.5	±	0.1
**TAG 18:2/18:3/18:3 (-18:2)**	0.3	±	0.0	0.3	±	0.1	0.6	±	0.2

Supplementary Table 9. Changes in molecular species of diacylglycerol (DAG) during seed development. Means ± s.d. (n=5).

	20 DAF			27 DAF			35 DAF		
**DAG 16:0/16:1**	0.5	±	0.1	0.1	±	0.0	0.1	±	0.0
**DAG 16:0/18:1**	4.4	±	0.4	2.3	±	0.1	2.2	±	0.1
**DAG 16:0/18:2**	10.4	±	0.6	4.2	±	0.9	3.2	±	0.4
**DAG 16:1/18:1**	0.9	±	0.2	0.4	±	0.1	0.4	±	0.0
**DAG 16:0/18:3**	2.1	±	0.1	0.9	±	0.2	1.1	±	0.1
**DAG 16:1/18:2**	0.6	±	0.1	0.4	±	0.1	0.4	±	0.0
**DAG 16:1/18:3**	0.1	±	0.0	0.1	±	0.0	0.1	±	0.0
**DAG 16:2/18:2**	0.1	±	0.0	0.1	±	0.0	0.1	±	0.0
**DAG 18:1/18:1**	29.5	±	0.9	34.3	±	5.9	39.3	±	2.5
**DAG 18:1/18:2**	29.1	±	0.7	38.4	±	1.1	36.2	±	1.6
**DAG 18:1/18:3**	5.7	±	0.2	6.1	±	0.6	7.4	±	0.5
**DAG 18:2/18:2**	9.9	±	0.5	8.8	±	3.0	6.8	±	0.8
**DAG 18:2/18:3**	6.6	±	0.3	3.6	±	1.1	2.7	±	0.3
**DAG 18:0/20:0**	0.0	±	0.0	0.0	±	0.0	0.0	±	0.0
**DAG 18:0/20:1**	0.0	±	0.0	0.0	±	0.0	0.0	±	0.0
**DAG 18:3/20:1**	0.0	±	0.0	0.1	±	0.0	0.1	±	0.0

Supplementary Table 10. Changes in molecular species of phosphatidic acid (PA) during seed development. Means ± s.d. (n=5).

	20 DAF			27 DAF			35 DAF		
**PA 16:0/16:1**	0.4	±	0.0	0.4	±	0.1	0.3	±	0.1
**PA 16:0/18:1**	4.2	±	0.3	3.8	±	0.3	4.8	±	1.0
**PA 16:1/18:1**	0.6	±	0.1	0.7	±	0.2	0.7	±	0.1
**PA 16:0/18:2**	21.5	±	0.8	11.6	±	1.4	11.5	±	1.0
**PA 16:0/18:3**	4.6	±	0.3	3.6	±	1.0	5.7	±	1.0
**PA 16:1/18:2**	1.1	±	0.1	1.1	±	0.4	1.0	±	0.2
**PA 16:1/18:3**	0.1	±	0.0	0.1	±	0.1	0.1	±	0.1
**PA 16:2/18:2**	0.1	±	0.0	0.1	±	0.1	0.0	±	0.0
**PA 16:2/18:3**	0.0	±	0.0	0.0	±	0.0	0.0	±	0.0
**PA 18:0/18:1**	1.6	±	0.3	1.6	±	0.2	1.2	±	0.2
**PA 18:1/18:1**	10.6	±	1.6	18.8	±	2.2	15.4	±	2.6
**PA 18:1/18:2**	17.2	±	1.1	26.3	±	1.2	27.8	±	1.4
**PA 18:1/18:3**	3.5	±	0.3	5.2	±	0.4	6.8	±	1.5
**PA 18:2/18:2**	24.8	±	2.0	16.9	±	1.7	13.4	±	1.1
**PA 18:2/18:3**	8.4	±	0.3	7.4	±	1.3	6.6	±	0.5
**PA 18:1/20:1**	0.1	±	0.0	0.3	±	0.1	0.2	±	0.1
**PA 18:2/20:1**	0.1	±	0.0	0.2	±	0.1	0.1	±	0.1
**PA 18:3/18:3**	1.1	±	0.1	2.0	±	0.8	4.3	±	0.8

Supplemental Table 11. Changes in molecular species of phosphatidylcholine (PC) during seed development. Means ± s.d. (n=5).

	20 DAF			27 DAF			35 DAF		
**PC 16:0/16:1**	0.5	±	0.3	0.5	±	0.4	0.4	±	0.1
**PC 16:0/18:1**	7.3	±	1.3	6.1	±	0.5	8.3	±	1.3
**PC 16:0/18:2**	16.1	±	1.8	8.6	±	1.0	7.9	±	1.7
**PC 16:1/18:1**	0.4	±	0.4	0.6	±	0.5	0.8	±	0.3
**PC 16:0/18:3**	3.6	±	1.1	2.6	±	1.2	1.9	±	0.7
**PC 16:1/18:2**	0.8	±	0.3	0.9	±	0.3	0.8	±	0.4
**PC 16:1/18:3**	0.1	±	0.1	0.1	±	0.1	0.2	±	0.2
**PC 16:2/18:2**	4.1	±	0.7	2.8	±	0.5	1.0	±	0.2
**PC 16:2/18:3**	1.4	±	0.5	2.1	±	0.6	1.3	±	0.3
**PC 18:0/18:1**	5.0	±	1.0	8.5	±	1.2	8.8	±	1.2
**PC 18:1/18:1**	10.9	±	2.2	24.6	±	2.2	23.2	±	2.9
**PC 18:1/18:2**	18.2	±	0.8	22.9	±	1.3	26.8	±	1.7
**PC 18:1/18:3**	3.9	±	0.6	3.4	±	0.6	5.1	±	1.2
**PC 18:2/18:2**	17.3	±	2.1	10.0	±	1.7	8.6	±	2.1
**PC 18:2/18:3**	8.8	±	1.6	4.8	±	0.9	3.7	±	0.7
**PC 18:3/18:3**	1.3	±	0.9	1.1	±	0.7	1.0	±	0.4
**PC 18:1/20:1**	0.0	±	0.1	0.4	±	0.3	0.0	±	0.0
**PC 18:2/20:1**	0.2	±	0.2	0.0	±	0.1	0.1	±	0.1

Supplemental Table 12. Changes in molecular species of phosphatidylethanolamine (PE) during seed development. Means ± s.d. (n=5).

	20 DAF	27 DAF	35 DAF
**PE 16:0/16:1**	0.5 ± 0.1	0.8 ± 0.1	0.3 ± 0.1
**PE 16:0/18:1**	3.7 ± 0.3	5.9 ± 1.2	6.1 ± 0.9
**PE 16:0/18:2**	35.2 ± 0.7	19.5 ± 2.9	18.0 ± 1.9
**PE 16:1/18:1**	0.4 ± 0.1	0.7 ± 0.1	0.6 ± 0.1
**PE 16:0/18:3**	6.4 ± 0.8	5.9 ± 0.7	4.3 ± 0.8
**PE 16:1/18:2**	1.2 ± 0.3	1.4 ± 0.3	0.9 ± 0.1
**PE 16:1/18:3**	0.1 ± 0.1	0.2 ± 0.0	0.2 ± 0.1
**PE 16:2/18:2**	0.1 ± 0.1	0.3 ± 0.2	0.1 ± 0.1
**PE 16:2/18:3**	0.0 ± 0.0	0.0 ± 0.0	0.0 ± 0.0
**PE 18:0/18:1**	1.3 ± 0.3	1.2 ± 0.4	1.1 ± 0.2
**PE 18:1/18:1**	4.4 ± 0.7	9.9 ± 1.8	11.0 ± 1.9
**PE 18:1/18:2**	14.5 ± 0.9	23.8 ± 2.4	29.7 ± 0.8
**PE 18:1/18:3**	3.4 ± 0.3	5.7 ± 0.7	5.1 ± 0.8
**PE 18:2/18:2**	22.2 ± 1.1	16.8 ± 2.2	17.3 ± 2.1
**PE 18:2/18:3**	4.7 ± 0.4	4.3 ± 0.3	2.7 ± 0.9
**PE 18:3/18:3**	1.3 ± 0.2	2.5 ± 0.3	1.9 ± 0.5
**PE 18:1/20:1**	0.2 ± 0.1	0.5 ± 0.3	0.5 ± 0.7
**PE 18:2/20:1**	0.3 ± 0.1	0.7 ± 0.5	0.2 ± 0.0

Supplemental Table 13. Changes in molecular species of acyl-CoA during seed development. Means ± s.d. (n=5).

	20 DAF			27 DAF			35 DAF		
**14:0**	4.05	±	0.76	6.04	±	0.96	4.13	±	0.52
**16:0**	11.54	±	2.26	15.25	±	3.93	8.49	±	3.42
**16:1**	0.54	±	0.13	1.21	±	0.15	1.58	±	0.71
**18:0**	6.11	±	1.15	6.86	±	1.51	6.64	±	0.92
**18:1**	21.12	±	1.60	45.62	±	4.64	78.03	±	10.56
**18:2**	13.39	±	1.55	18.03	±	1.87	26.21	±	3.00
**18:3**	5.69	±	0.77	9.48	±	1.63	9.93	±	0.82
**20:0**	1.93	±	0.45	3.52	±	0.73	1.35	±	0.49
**20:1**	1.22	±	0.61	2.20	±	0.29	0.42	±	0.23
**22:0**	1.13	±	0.47	3.12	±	1.35	0.47	±	0.28
**22:1**	0.85	±	0.21	2.23	±	0.29	1.89	±	1.00
**24:0**	5.35	±	0.52	4.87	±	0.80	2.14	±	0.85
**24:1**	0.79	±	0.13	3.04	±	1.27	0.82	±	0.40
**26:0**	2.14	±	0.36	3.53	±	1.13	0.56	±	0.28
**26:1**	0.52	±	0.10	0.97	±	0.45	0.19	±	0.09
**28:0**	1.19	±	0.18	1.08	±	0.27	0.15	±	0.06
**28:1**	1.03	±	0.22	1.63	±	0.59	0.37	±	0.18
**30:0**	0.80	±	0.26	0.51	±	0.16	0.08	±	0.03
**30:1**	1.12	±	0.34	1.32	±	0.47	0.18	±	0.05
